# Temporal Variation of Litterfall and Nutrient Return of *Serianthes nelsonii* Merr. in a Tropical Karst Forest

**DOI:** 10.3390/plants11172310

**Published:** 2022-09-03

**Authors:** Thomas E. Marler, Gil N. Cruz

**Affiliations:** 1 Bagong Kaalaman Botanikal Institute, 15 Rizal Street, Barangay Malabañas, Angeles City 2009, Philippines; 2 Western Pacific Tropical Research Center, University of Guam, Mangilao, GU 96923, USA

**Keywords:** decomposition, Guam, nutrient flux

## Abstract

Trees contribute to ecosystem nutrient cycling through the amount, timing, and composition of litterfall. Understanding the nature of this contribution from endangered tree species may aid in species and habitat recovery efforts. *Serianthes nelsonii* is an endangered tree species from the Mariana Islands, and little is known about litterfall dynamics. The timing of leaf, fruit, and stem litterfall was determined to more fully understand the return of nutrients via litter. The total annual litterfall was 272.8 g·m^−2^, with 45% represented by leaves, 48% represented by stems, and 7% represented by fruits. Stem litterfall weight contrasted more from month to month than the other organs, and leaf litterfall exhibited the most even distribution throughout the year. The timing of fruit and stem litterfall was influenced by the timing of extreme wind events. Leaf litter contributed nutrients in the following order: carbon > calcium > nitrogen > potassium > magnesium > iron > phosphorus > manganese > boron > zinc > copper. Fruit and stem litter contributed nutrients in the following order: carbon > calcium > nitrogen > magnesium > potassium > phosphorus > iron > manganese > boron > zinc > copper. Based on carbon/nitrogen, the stem litter exhibited the lowest quality and leaf litter exhibited the highest quality for speed of nutrient release via decomposition. Conservationists may use this knowledge to more fully integrate *S. nelsonii* trees into habitat management plans.

## 1. Introduction

Nutrient return to soil through plant litter is a defining aspect of nutrient cycling and soil food web dynamics in forested ecosystems. Plant litter plays an important role in sustaining soil fertility, the global carbon (C) cycle, and soil biodiversity [1]. Most research on this topic has focused on litter and soil characteristics that predict decomposition speed, but Killingbeck [2] noted that litter nutrient concentrations may differ over time and attributed these temporal changes to variations of resorption efficiency and proficiency. Although limited in scope, several studies have discussed how the time of year influences litter nutrient deposition from plants to soils [3,4,5,6,7,8,9,10,11,12,13,14].

The prescribed planting of native trees in urban and peri-urban systems and in restoration plantings is a promising way to manage litter deposition and nutrient cycling and foster recovery of degraded areas. Recent initiatives are coordinating efforts among many agencies to improve Guam forest health and resilience with increased plantings of native plant species [15]. *Serianthes nelsonii* is a critically endangered Fabaceae tree [16,17], and is among the native plant species that are actively being planted to address the goals. However, to orient the selection of which trees to use, there is a need expand knowledge about the potential of native trees in producing litter and contributing nutrients to the ecosystem. Several recent reports have included leaf nutrient traits for *S. nelsonii* [18,19,20], but nothing has been published concerning the seasonal variations of fresh or senesced leaves and other organs. Therefore, a better understanding of how nutrient content changes throughout the year is needed to appreciate the ecosystem services provided by *S. nelsonii* and factor this information into habitat management plans.

This study aimed to evaluate monthly production and nutrient return to soils of leaf, fruit, and stem litter from a mature *S. nelsonii* tree in northern Guam. The objectives were to determine the influence of months on *S. nelsonii* litter quantity and quality to better understand the contributions of this species to nutrient flux in the tropical karst ecosystem.

## 2. Materials and Methods

The tropical wet climate of the study site in northern Guam is Af under the Köppen-Geiger classification. The karst soils that support the forests in the coastal zones of northern Guam developed in slope alluvium, loess, and residuum overlying limestone (Clayey-skeletal, gibbsitic, nonacid, isohyperthermic Lithic Ustorthents) [21]. Litterfall dynamics were studied beneath the only known mature *S. nelsonii* tree in Guam, and the tree exhibited a diameter at breast height of ca. 56 cm and maximum canopy radius of 4.8 m.

Litterfall beneath the tree was collected using six litter traps that were 50 cm in diameter and installed about 20 cm above the forest floor. The collections began in late 2012 and continued through early 2014. The details of the site and collection methods were described previously [22]. For the purpose of this study, we pooled all litter collected during each month throughout 2013. The collection dates occurred in the first and third week of each month, so the pooled litter reported for each month included the second half of the previous month and the first half of the current month. Processing the samples began with removal of all litter components that were not from the *S. nelsonii* tree. The remaining *S. nelsonii* litter was separated into leaf, fruit, and stem litter. Litter from flowers was mostly abscised stamens and the volume was negligible and rarely recovered in the traps. When found, we added the flower litter to the fruit litter samples. Seeds within the fruits were extracted and either used in recovery plantings or returned to the seed bank, therefore there was no removal of propagules or any living materials during the course of this study.

For each month, the tissue from three organ types in each of six traps was dried in a forced draft oven for 48 h at 75 °C. The weight of each sample was measured. Some of the individual trap samples did not contain enough tissue volume for the elemental analysis of the litter. Therefore, the tissue of each organ type from all six traps was combined into one sample for each month. The tissue was milled to pass through 20-mesh screen. Total nitrogen (N) and total C were determined by dry combustion [23] (LECO CN Analyzer, LECO Corporation, St. Joseph, MI, USA). Litter minerals and metals were digested with diethylene triamine pentaacetic acid and were quantified by inductively coupled plasma optical emission spectrometry [24] (Spectro Genesis; SPECTRO Analytical Instruments, Kleve, Germany). Elements included in the analysis were phosphorus (P), potassium (K), calcium (Ca), magnesium (Mg), manganese (Mn), iron (Fe), zinc (Zn), boron (B), and copper (Cu).

The concentrations of the minerals and metals were used to calculate the estimated pool of each element in each litter trap to provide six replications per month for the total tissue dry weight and the total weight of each mineral and metal. For this purpose, the weights per trap were converted to g, mg, or µg per square meter. The data did not conform to prerequisites for use of parametric analytical methods. Therefore, the data were subjected to the Kruskal–Wallis *H* test (SAS Institute, Cary, NC, USA) to determine differences among the 12 months for each of the response variables. A variability index (VI) was calculated for each response variable as (monthly maximum − monthly minimum)/monthly maximum.

## 3. Results

### 3.1. Leaf Litter

**Figure 1:**
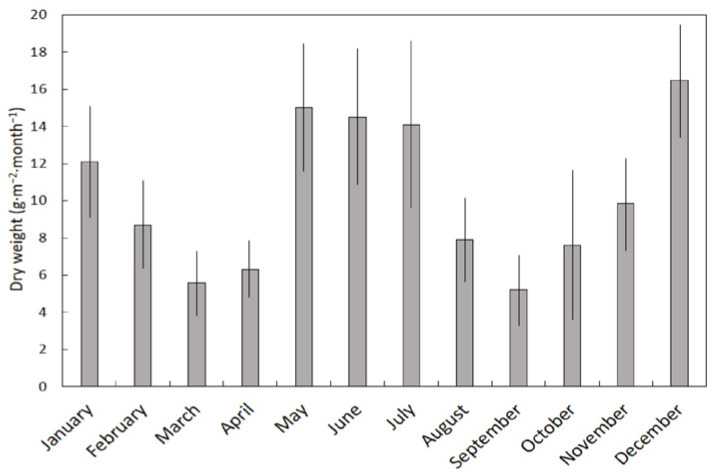
The influence of month on leaf litterfall beneath a *Serianthes nelsonii* tree. Mean ± SE, *n* = 6.

The dry weight of leaf litterfall was highly contrasting among the months (*H* = 20.96, *p* = 0.034). March, April, and September were the months with the least leaf litterfall, and May, June, July, and December were the months with the greatest leaf litterfall (Figure 1). The VI for leaf litterfall was 0.68.

The macronutrients contained within the monthly leaf litter pools were highly contrasting among the elements and months (Table 1). The leaf litter inputs of C paralleled those of litter mass, as almost half of the leaf litter comprised C. The monthly mean was 4631 mg·m^−2^, and there was a 3.1-fold range in C among the months. The VI for N and P were similar to that of C, but the differences between the months were not significant. Leaf N averaged 174 mg·m^−2^, and leaf P averaged 10 mg·m^−2^. Leaf K was the most variable macronutrient, with a minimum in April and a maximum in December. Leaf Ca was the most abundant element in the litter after C, and the monthly mean was 310 mg·m^−2^. Leaf Mg pattern was unique, with a minimum in September and a maximum in May.

The micronutrients contained within the leaf litterfall were also contrasting among the months (Table 2). The leaf Zn content did not differ among the various months and averaged 197 µg·m^−2^. The Mn content of leaf litter was least in March and most in December. Leaf Fe content was greater than the other micronutrients, with a monthly mean of 13,774 µg·m^−2^ and a VI that exceeded that of all other elements. Leaf Cu was minimal, with a monthly mean of only 25 µg·m^−2^. Leaf B averaged 618 µg·m^−2^ and exhibited a minimum in September and a maximum in May.

### 3.2. Fruit Litter

**Figure 2:**
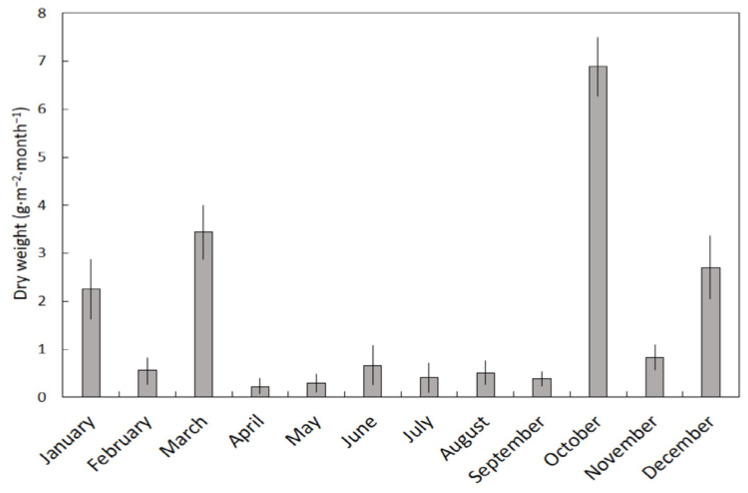
The influence of month on fruit litterfall beneath a *Serianthes nelsonii* tree. Mean ± SE, *n* = 6.

The dry weight of fruit litterfall varied greatly among the months (*H* = 33.14, *p* < 0.001). April and May were the months with the least fruit litterfall, and October was the month with the greatest fruit litterfall (Figure 2). The VI for fruit litterfall was 0.97, greatly exceeding that of leaf litter.

The macronutrients contained within the monthly fruit litter pools were highly variable among the elements and months (Table 3). The VI and level of significance for macronutrients in the fruit litterfall exceeded the VI and the levels of significance of macronutrients in leaf litterfall. Moreover, the general ranking among the months was similar for each of the macronutrients in fruit litter. The litter collected in October consistently contained more of each macronutrient than any other month, and the litter collected in April contained less of each macronutrient than any other month. Monthly leaf litter inputs of C were greater than for the other macronutrients with a mean of 691 mg·m^−2^, and monthly leaf litter inputs of P were less than for the other macronutrients with a mean of only 0.7 mg·m^−2^.

The micronutrients contained within the fruit litterfall were also contrasting among the months in manner similar to that of macronutrients (Table 4). The fruit litter VI and level of significance for micronutrients among the months greatly exceeded the micronutrient variability in leaf litterfall. The most abundant micronutrient in the fruit litter was Fe with a monthly mean of 53 µg·m^−2^, and the least abundant micronutrient in fruit litter was Cu with a monthly mean of only 2 µg·m^−2^. 

### 3.3. Stem Litter

**Figure 3:**
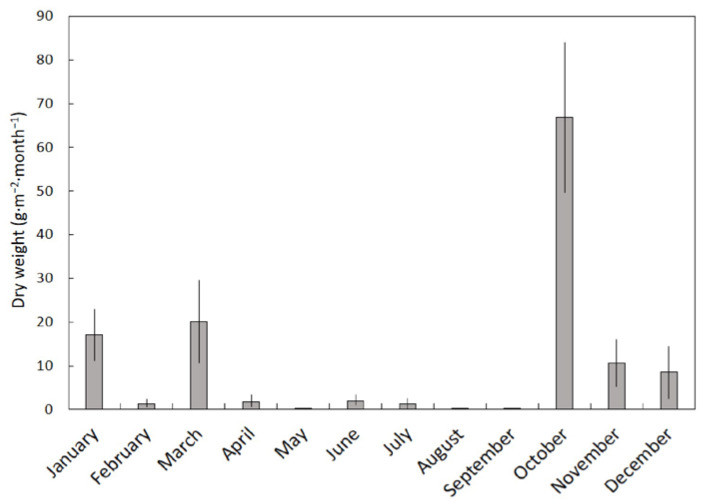
The influence of month on stem litterfall beneath a *Serianthes nelsonii* tree. Mean ± SE, *n* = 6.

The variation of stem litterfall dry weight was much more variable among the months than leaf or fruit litterfall (*H* = 46.70, *p* < 0.001). Stem litter was minimal for February and the months from April until September (Figure 3). Substantial stem litterfall was recorded for October due to a high wind event. The VI for stem litterfall was 0.99.

The variation among the months for macronutrient inputs within stem litter pools was greater than for leaf or fruit litter (Table 5). As with fruit litter, the stem litter collected in October consistently contained more of each macronutrient than any other month. In contrast with fruit litter, the month with the least amount of macronutrient input in stem litter varied among the elements. Monthly leaf litter inputs of C were greater than for the other macronutrients with a mean of 4620 mg·m^−2^, and monthly leaf litter inputs of P were less than for the other macronutrients with a mean of only 3.9 mg·m^−2^.

The patterns of micronutrient input within stem litter were similar to those of the macronutrients, with the monthly variation exceeding that of leaf and fruit litter (Table 6). The most abundant micronutrient in the fruit litter was Fe with a monthly mean of 420 µg·m^−2^, and the least abundant micronutrient in fruit litter was Cu with a monthly mean of 18 µg·m^−2^.

### 3.4. Annual Litter Quality Relations

Total annual dry weight of the *S. nelsonii* leaf litter was 1234 kg·ha^−1^, of the fruit litter was 191 kg·ha^−1^, and of the stem litter was 1303 kg·ha^−1^. Annual stem litter dry weight exceeded that of leaf litter despite the fact that the stem litterfall was negligible in seven months of the year. This was due to litterfall in the month of October. The ranking of nutrient inputs from leaf litter varied slightly between some of the elements, especially for the months with the least litterfall. However, when the data were pooled for the entire year, the *S. nelsonii* tree returned essential nutrients to the soil via leaf litter in the following order: C > Ca > N > K > Mg > Fe > P > Mn > B > Zn > Cu. The variation in ranking of nutrients among the months was similar for fruit and stem litter, so the variation in tissue dry weight was the controlling factor in the monthly variation of nutrient inputs. When the data were pooled for the entire year, the *S. nelsonii* tree returned essential nutrients to the soil via fruit and stem litter in the following order: C > Ca > N > Mg > K > P > Fe > Mn > B > Zn > Cu. 

Litter quality, as defined by C/N, was dissimilar among the three litter types. The most labile litter was leaf litter with a mean C/N of 26.6. The most recalcitrant litter was stem litter with a mean C/N of 45.0. Fruit litter was intermediate in quality with a mean C/N of 34.5.

## 4. Discussion

Nutrient return to soil through plant litter plays an important role in sustaining soil fertility and many aspects of ecosystem health [1]. One inadequately studied aspect of litter production is the temporal dynamics of litterfall variation [2,3,4,5,6,7,8,9,10,11,12,13,14]. Highly contrasting monthly litterfall is often correlated to growing versus non-growing seasons based on temperatures [4,9,11,12] or rainy versus dry seasons [5,6,7,13,14]. The benign climate of the Mariana Islands is not characterized by strong seasonal patterns. January–May comprises the dry season, and July–November comprises the rainy season. The leaf litterfall we report herein did not differ between the two seasons, but the stem and fruit litterfall was greater in the rainy season. As a result, 949 kg·ha^−1^ of *S. nelsonii* litterfall occurred in the dry season and 1329 kg·ha^−1^ occurred in the rainy season. However, these results were caused by stochastic high wind events rather than rainfall patterns per se. The strongest winds during the study period occurred on 20 September 2013 with maximum winds of 84 km·h^−1^, and the litterfall that resulted from this wind event was collected during the first of our October collection dates. This one wind event caused the October collection dates to account for 51% of the annual stem litter and 36% of the annual fruit litter. The seasonal patterns of litterfall in Pohnpei, a second island in Micronesia, exhibited a similar increase in litterfall during the rainy season, but the causes were not discussed [14].

Our total litter inputs for the year accumulated to 2728 kg·ha^−1^, an amount that was less than reported for other tropical regions e.g., [14,25,26,27,28]. However, our methods were unique, in that we focused on litterfall from the single species *S. nelsonii*, and therefore removed the litter that originated from other sympatric tree species. The dry weight of the non-*Serianthes* litter that was collected from our traps was 4435 kg·ha^−1^, indicating total annual litterfall accumulated to 7163 kg·ha^−1^. This total litterfall was more in congruence with the earlier reports from tropical forests [14,25,26,27,28]. We did not separate the non-*Serianthes* litter of the other tree species. However, common tree species in the habitat included *Aglaia mariannensis* Merr., *Eugenia reinwardtiana* (Blume) DC., *Ficus prolixa* G.Forst., *Macaranga thompsonii* Merr., *Meiogyne cylindrocarpa* (Burck) Heusden, *Ochrosia mariannensis* A.DC., and *Ochrosia oppositifolia* (Lam.) K.Schum. 

The past and ongoing research into the agenda of forest ecosystem services has included C and N as high priority topics [29]. The relative proportion of these two elements in litter is one of the more important traits used to predict litter decomposition speed [30,31]. In turn, decomposition speed operates in concert with litterfall quantity and timing to define the volume of standing litter on the forest floor. The spatial heterogeneity of standing litter volume has direct impact on many aspects of forest ecology [32] and creates different regeneration niches, which contribute to species diversity [33,34]. Litter layer integrity may also influence localized soil nutrition by reducing the risk of erosion [35]. In this study, the predicted speed of decomposition of *S. nelsonii* litter based on C/N was in the order wood < fruit < leaf. The relatively even timing of labile leaf litterfall throughout the year contrasted sharply with the pulsed timing of recalcitrant wood litter inputs, and the interplay of these behaviors may be part of the controlling factors that define the spatiotemporal characteristics of the litter layer in this tropical karst forest.

Environmental factors interact with litter quality to control the speed of plant litter decomposition. Among these factors are the amount and timing of rainfall. Indeed, decomposition speed is more rapid during high rainfall seasons than low rainfall seasons [36,37,38,39,40]. Developing a full understanding of how *S. nelsonii* participates in the nutrient flux of this tropical karst forest will require more research on seasonal aspects of actual litter decomposition to augment our results which focused on litter inputs. The unique characteristics of *S. nelsonii* litter decomposition may help to better understand the means by which the tree creates unique biogeochemical spatial niches [41].

The forests in the Mariana Islands experience more tropical cyclones than in any other state or territory of the United States [42]. These extreme wind events occur on Guam with such frequency [43] that they must be factored into ecosystem management decisions. Climate change models predict a greater intensity of tropical cyclones in the future [44]. Our findings that ephemeral high wind events are a dominant factor controlling the annual litterfall cycle in this tropical karst forest indicate that the predicted changes in tropical cyclone intensity may impose extreme changes in nutrient flux in the region.

The litter component that was dominant in our study was stem tissue, causing leaf litter to account for only 45% of the annual litterfall. Leaf tissue accounts for about 70% of litterfall in most forests globally [45]. We suggest that the frequency of tropical cyclones and extreme wind events in the Mariana Islands is responsible for the greater representation of stem tissue in this Guam forest litter. These observations illuminate the need for more litterfall studies to determine if all forest habitats in the Mariana Islands exhibit increased stem tissue and decreased leaf tissue in litterfall relative to global patterns.

Extinction risk for more than half of the world’s threatened species may not be addressed adequately unless species-specific recovery plans are developed by knowledgeable conservationists [46]. Against this backdrop, efforts to safeguard tree diversity from continued threats has become a global challenge [47,48]. Addressing this challenge will demand the use of adaptive management approaches, whereby non-destructive experimental and monitoring approaches that generate new evidence are integrated into programming [49]. In support of these global efforts, federal agencies are attempting to coordinate efforts to improve Guam’s forest health and resilience by increasing native plant coverage [15]. *Serianthes nelsonii* is among the list of native plant species that are actively being planted to address this initiative. The 28-year-old national recovery plan for this tree species [50] has been inadequately implemented, partly due to historical choices to ignore the value of funding adaptive management research conducted by competent scientists to generate new knowledge [51]. The failures of meeting the goals of the recovery plan reveal how past decision makers can create a level of distrust that impedes continued recovery efforts, particularly where militarization is part of the conservation model, such as the case in Guam [52]. Our methods provide an example of how non-destructive methods may be used by practitioners to generate new evidence that can be factored into conservation programming to inform species and habitat recovery plans. This snapshot of *S. nelsonii* litterfall behaviors in 2013 also provides a benchmark for future studies to use repeatable methods to quantify alterations in litterfall that accompany future climate change [53].

## Figures and Tables

**Table 1:** The influence of month of year on macronutrient return (mg·m^−2^) in *Serianthes nelsonii* leaf litter.

Month	Carbon	Nitrogen	Phosphorus	Potassium	Calcium	Magnesium
January	5365 ± 993	229.6 ± 47.2	14.5 ± 3.0	58.0 ± 11.9	350.4 ± 72.1	49.5 ± 10.2
February	3863 ± 828	171.8 ± 36.8	12.2 ± 2.6	55.8 ± 12.0	269.5 ± 57.8	40.1 ± 8.6
March	2432 ± 341	99.4 ± 13.9	7.2 ± 1.1	38.3 ± 5.4	160.0 ± 22.4	24.4 ± 3.4
April	2812 ± 603	101.8 ± 21.8	6.3 ± 1.4	29.1 ± 6.2	201.6 ± 43.2	29.1 ± 6.2
May	6840 ± 1381	213.4 ± 43.1	12.0 ± 2.4	67.7 ± 13.7	441.9 ± 89.2	61.6 ± 12.4
June	6608 ± 1655	215.0 ± 53.8	13.1 ± 3.3	69.7 ± 17.5	431.4 ± 108.0	56.6 ± 14.2
July	6426 ± 2112	211.9 ± 69.6	11.3 ± 3.7	43.8 ± 14.4	436.4 ± 143.4	53.7 ± 17.6
August	3616 ± 1268	116.6 ± 40.9	6.3 ± 2.2	27.0 ± 9.5	255.3 ± 89.6	28.5 ± 10.1
September	2357 ± 778	100.8 ± 33.3	5.7 ± 1.9	16.5 ± 5.5	158.1 ± 52.2	17.1 ± 5.6
October	3494 ± 1769	151.4 ± 76.6	8.4 ± 4.3	21.4 ± 10.8	223.3 ± 113.0	24.5 ± 12.4
November	4414 ± 1121	176.4 ± 44.8	9.9 ± 2.5	40.4 ± 10.3	304.5 ± 77.3	36.5 ± 9.3
December	7337 ± 1488	304.3 ± 61.7	18.1 ± 3.7	70.7 ± 14.3	493.5 ± 100.1	57.6 ± 11.7
*H*	21.531	18.451	19.521	27.201	20.493	21.234
*p*	0.028	0.072	0.052	0.004	0.039	0.031
VI ^1^	0.68	0.67	0.69	0.77	0.68	0.72

^1^ Variability Index = (monthly maximum − monthly minimum)/monthly maximum.

**Table 2:** The influence of month of year on micronutrient return (µg·m^−2^) in *Serianthes nelsonii* leaf litter.

Month	Zinc	Manganese	Iron	Copper	Boron
January	277.9 ± 57.1	978.8 ± 201.3	14,329 ± 5264	24.2 ± 5.0	712.9 ± 146.6
February	305.2 ± 65.5	837.1 ± 179.5	8979 ± 3556	26.2 ± 5.6	514.5 ± 110.3
March	116.6 ± 16.3	466.6 ± 65.4	2846 ± 712	16.7 ± 2.3	338.8 ± 47.5
April	107.5 ± 23.0	486.7 ± 104.3	3783 ± 1388	12.6 ± 2.7	474.0 ± 101.6
May	180.4 ± 36.4	1157.6 ± 233.7	20,950 ± 8366	45.1 ± 9.1	1112.4 ± 224.6
June	174.3 ± 43.7	1249.2 ± 312.8	23,831 ± 11,523	29.1 ± 7.3	886.0 ± 221.8
July	183.6 ± 60.3	1228.7 ± 403.8	26,724 ± 12,659	42.3 ± 13.9	833.3 ± 273.8
August	118.9 ± 41.7	674.0 ± 236.4	8632 ± 4682	23.8 ± 8.3	459.9 ± 161.3
September	128.8 ± 39.2	485.8 ± 160.3	3877 ± 1845	10.3 ± 3.4	211.9 ± 69.9
October	298.2 ± 150.9	611.6 ± 309.7	10,670 ± 7259	22.9 ± 11.6	328.8 ± 166.5
November	197.1 ± 50.0	867.1 ± 220.2	11,297 ± 4861	19.7 ± 5.0	472.9 ± 120.1
December	279.7 ± 56.7	1480.6 ± 300.2	29,364 ± 11,823	32.9 ± 6.7	1069.3 ± 216.8
*H*	18.962	21.544	21.378	22.992	38.519
*P*	0.062	0.028	0.030	0.018	<0.001
VI ^1^	0.65	0.68	0.90	0.77	0.81

^1^ Variability Index = (monthly maximum − monthly minimum)/monthly maximum.

**Table 3:** The influence of month of year on macronutrient return (mg·m^−2^) in *Serianthes nelsonii* fruit litter.

Month	Carbon	Nitrogen	Phosphorus	Potassium	Calcium	Magnesium
January	960 ± 354	29.9 ± 11.0	1.1 ± 0.4	3.4 ± 1.2	64.2 ± 23.7	2.8 ± 1.7
February	244 ± 114	7.1 ± 3.3	0.3 ± 0.1	0.8 ± 0.4	16.8 ± 7.8	1.8 ± 0.6
March	1475 ± 432	40.7 ± 11.9	1.7 ± 0.5	6.2 ± 2.0	104.4 ± 30.6	6.2 ± 1.8
April	94 ± 94	2.5 ± 2.5	0.1 ± 0.1	0.3 ± 0.3	6.2 ± 6.2	0.4 ± 0.4
May	128 ± 114	3.9 ± 2.4	0.1 ± 0.1	0.4 ± 0.3	8.5 ± 7.5	0.5 ± 0.5
June	285 ± 285	7.8 ± 7.8	0.3 ± 0.3	0.9 ± 0.9	18.8 ± 18.8	1.9 ± 1.9
July	176 ± 176	4.8 ± 4.8	0.2 ± 0.2	0.5 ± 0.5	11.6 ± 11.6	0.7 ± 0.7
August	215 ± 215	5.8 ± 5.8	0.3 ± 0.3	0.7 ± 0.7	14.2 ± 14.2	0.9 ± 0.9
September	168 ± 168	4.5 ± 4.5	0.2 ± 0.2	0.5 ± 0.5	11.1 ± 11.1	0.7 ± 0.7
October	3036 ± 620	82.6 ± 16.9	2.8 ± 0.6	6.9 ± 1.3	163.2 ± 33.3	12.4 ± 2.5
November	357 ± 230	11.9 ± 7.7	0.5 ± 0.3	1.9 ± 1.2	19.3 ± 12.4	1.7 ± 1.1
December	1148 ± 636	38.4 ± 21.2	1.3 ± 0.7	5.6 ± 3.1	61.9 ± 34.3	5.4 ± 2.9
*H*	33.014	33.488	32.169	31.13	34.316	32.206
*p*	<0.001	<0.001	0.001	0.001	<0.001	<0.001
VI ^1^	0.97	0.97	0.96	0.96	0.96	0.97

^1^ Variability Index = (monthly maximum − monthly minimum)/monthly maximum.

**Table 4:** The influence of month of year on micronutrient return (µg·m^−2^) in *Serianthes nelsonii* fruit litter.

Month	Zinc	Manganese	Iron	Copper	Boron
January	15.8 ± 5.8	87.9 ± 32.4	99.2 ± 36.5	2.3 ± 0.8	38.3 ± 14.1
February	3.4 ± 1.6	18.7 ± 8.7	17.5 ± 8.1	0.6 ± 0.3	9.0 ± 4.2
March	20.7 ± 6.1	110.3 ± 32.3	96.5 ± 28.3	6.9 ± 2.0	44.8 ± 13.1
April	1.3 ± 1.3	7.6 ± 7.6	6.3 ± 6.3	0.2 ± 0.2	2.4 ± 2.4
May	2.1 ± 1.9	10.1 ± 9.0	8.3 ± 7.4	0.3 ± 0.3	3.6 ± 3.2
June	4.0 ± 4.0	23.1 ± 23.1	19.2 ± 19.2	1.3 ± 1.3	7.3 ± 7.3
July	2.4 ± 2.4	13.9 ± 13.9	11.8 ± 11.8	0.4 ± 0.4	4.5 ± 4.5
August	3.5 ± 3.5	17.0 ± 17.0	13.5 ± 13.5	0.5 ± 0.5	6.5 ± 6.5
September	2.7 ± 2.7	13.3 ± 13.3	10.6 ± 10.6	0.4 ± 0.4	5.1 ± 5.1
October	41.3 ± 8.4	213.4 ± 43.6	213.4 ± 43.6	6.9 ± 1.4	96.4 ± 19.7
November	5.8 ± 3.7	24.8 ± 16.0	36.4 ± 23.5	0.8 ± 0.5	14.1 ± 9.1
December	16.1 ± 8.9	83.2 ± 46.1	107.3 ± 59.4	5.4 ± 3.0	42.9 ± 23.8
*H*	32.553	32.984	33.999	32.304	33.813
*p*	<0.001	<0.001	<0.001	<0.001	<0.001
VI ^1^	0.97	0.96	0.97	0.97	0.98

^1^ Variability Index = (monthly maximum − monthly minimum)/monthly maximum.

**Table 5:** The influence of month of year on macronutrient return (mg·m^−2^) in *Serianthes nelsonii* stem litter.

Month	Carbon	Nitrogen	Phosphorus	Potassium	Calcium	Magnesium
January	7339 ± 2629	171.5 ± 61.4	6.9 ± 2.5	24.0 ± 8.6	761.3 ± 272.7	36.0 ± 12.9
February	521 ± 355	14.7 ± 10.1	0.8 ± 0.5	2.6 ± 1.8	62.0 ± 42.3	3.2 ± 2.1
March	8606 ± 4039	189.0 ± 88.7	10.1 ± 4.7	34.2 ± 16.0	894.8 ± 419.9	40.2 ± 18.9
April	739 ± 449	16.9 ± 10.3	0.5 ± 0.3	2.5 ± 1.5	95.6 ± 58.1	3.9 ± 2.4
May	47 ± 47	1.1 ± 1.1	0.1 ± 0.1	0.2 ± 0.2	5.6 ± 5.6	0.3 ± 0.3
June	835 ± 528	19.7 ± 12.4	0.6 ± 0.4	3.0 ± 1.9	100.4 ± 63.5	4.8 ± 3.0
July	553 ± 553	13.2 ± 13.2	0.5 ± 0.5	2.1 ± 2.1	66.6 ± 66.6	3.0 ± 3.0
August	93 ± 93	2.2 ± 2.2	0.1 ± 0.1	0.4 ± 0.4	11.0 ± 11.0	0.5 ± 0.5
September	108 ± 108	2.6 ± 2.6	0.1 ± 0.1	0.5 ± 0.5	13.1 ± 13.1	0.6 ± 0.6
October	28,505 ± 7544	622.3 ± 164.7	20.1 ± 5.3	53.5 ± 14.2	3138.2 ± 517.6	133.8 ± 35.4
November	4489 ± 1689	93.4 ± 35.1	3.2 ± 1.2	9.6 ± 3.6	482.8 ± 181.7	24.4 ± 9.2
December	3616 ± 1188	87.0 ± 28.6	3.4 ± 1.1	12.9 ± 4.2	407.3 ± 133.8	20.7 ± 6.8
*H*	46.701	46.693	46.072	45.340	46.133	46.231
*p*	<0.001	<0.001	<0.001	<0.001	<0.001	<0.001
VI ^1^	0.99	0.99	0.99	0.99	0.99	0.99

^1^ Variability Index = (monthly maximum − monthly minimum)/monthly maximum.

**Table 6:** The influence of month of year on micronutrient return (µg·m^−2^) in *Serianthes nelsonii* stem litter.

Month	Zinc	Manganese	Iron	Copper	Boron
January	102.9 ± 36.9	411.5 ± 147.4	497.3 ± 178.1	17.1 ± 6.1	360.1 ± 129.0
February	10.1 ± 6.9	27.7 ± 18.9	46.6 ± 31.8	1.3 ± 0.9	22.7 ± 15.5
March	140.8 ± 66.1	482.6 ± 226.5	784.2 ± 368.0	40.2 ± 18.9	361.9 ± 169.9
April	3.6 ± 2.2	46.2 ± 28.1	71.1 ± 43.2	1.8 ± 1.1	37.3 ± 22.7
May	0.7 ± 0.7	3.5 ± 3.5	3.7 ± 3.7	0.2 ± 0.2	2.1 ± 2.1
June	11.9 ± 7.5	67.6 ± 42.7	73.5 ± 46.5	2.0 ± 1.3	37.8 ± 23.9
July	9.3 ± 9.3	42.3 ± 42.3	50.2 ± 50.2	2.6 ± 2.6	23.7 ± 23.7
August	1.8 ± 1.8	7.5 ± 7.5	7.7 ± 7.7	0.2 ± 0.2	4.2 ± 4.2
September	2.0 ± 2.0	8.7 ± 8.7	8.9 ± 8.9	0.3 ± 0.3	4.7 ± 4.7
October	468.4 ± 123.9	1271.3 ± 336.5	2743.4 ± 726.1	133.8 ± 35.4	1003.7 ± 265.6
November	42.4 ± 15.9	159.2 ± 59.9	445.7 ± 167.7	10.6 ± 4.0	169.8 ± 63.9
December	51.7 ± 17.0	163.6 ± 53.8	310.0 ± 101.9	8.6 ± 2.8	172.2 ± 56.6
*H*	46.799	44.238	46.381	46.799	46.716
*P*	<0.001	<0.001	<0.001	<0.001	<0.001
VI ^1^	0.99	0.99	0.99	0.99	0.99

^1^ Variability Index = (monthly maximum − monthly minimum)/monthly maximum.

## Data Availability

Data available upon request.
